# Correlates of critical illness-related encephalopathy predominate postmortem COVID-19 neuropathology

**DOI:** 10.1007/s00401-020-02213-y

**Published:** 2020-08-26

**Authors:** Nikolaus Deigendesch, Lara Sironi, Michael Kutza, Sven Wischnewski, Vidmante Fuchs, Jürgen Hench, Angela Frank, Ronny Nienhold, Kirsten D. Mertz, Gieri Cathomas, Matthias S. Matter, Martin Siegemund, Markus Tolnay, Lucas Schirmer, Anne-Katrin Pröbstel, Alexandar Tzankov, Stephan Frank

**Affiliations:** 1 Pathology, Institute of Medical Genetics and Pathology, University Hospital Basel, University of Basel, Schönbeinstrasse 40, 4031 Basel, Switzerland; 2 Department of Neurology and Mannheim Center for Translational Neuroscience, Medical Faculty Mannheim, University of Heidelberg, Mannheim, Germany; 3 Departments of Medicine and Biomedicine, Neurologic Clinic and Policlinic, University Hospital Basel, University of Basel, Basel, Switzerland; 4 Institute of Pathology, Cantonal Hospital Baselland, Liestal, Switzerland; 5 Department of Intensive Care, University Hospital, University of Basel, Basel, Switzerland; 6 Department of Clinical Research, University of Basel, Basel, Switzerland

Infections with severe acute respiratory syndrome coronavirus 2 (SARS-CoV-2) primarily lead to upper respiratory tract infection and its sequelae frequently dominate the clinical course of COVID-19 [11, 25]. In addition to the lung, various other organs such as kidneys, gut, and heart can be affected [13, 20, 25]. Initially less noticed, it is now well documented that patients with COVID-19 can clinically present with a variety of neurological symptoms ranging from anosmia and dysgeusia to headache, impaired consciousness, agitation, and corticospinal tract signs [14]. Moreover, COVID-19 patient presentations with acute ischemic stroke, meningoencephalitis, hemorrhagic posterior reversible encephalopathy syndrome, acute disseminated encephalomyelitis (ADEM)-like pathology, as well as with diffuse leukoencephalopathy and microhemorrhages are on record [13, 21, 22]. Despite this wide range of neurological affections, it has so far remained unclear whether the reported abnormalities are pathogenetically linked to SARS-CoV-2 or occur coincidentally or in association with critical illness.

Here, we report on autoptic neuropathological findings for a study cohort of seven COVID-19 patients, all of whom were positive for SARS-CoV-2 by nasopharyngeal swab testing, and compare our observations with those made in a SARS-CoV-2 negative control autopsy cohort comprising individuals with non-septic and systemic inflammatory/septic clinical courses (Suppl. Tables 1—3). All patients of our study cohort except for one had multiple relevant comorbidities, consistent with the previous reports [11, 12, 16].

All COVID-19 study patients showed strong systemic inflammation, documented by high plasma levels of acute-phase proteins (i.e., fibrinogen, C-reactive protein), and interleukin 6. Systemic immune activation affects the CNS, resulting in so-called sickness behavior including lethargy, malaise, and fatigue [8]. As microglia, resident phagocytes of the CNS, are believed to contribute to this phenotype [26], we hypothesized that their activation contributes to the neurological phenotype of COVID-19. We chose HLA-DR, an MHC class II antigen, as surrogate marker for microglia activation [15, 26]. We found microglia activation in the brainstem of COVID-19 patients to be significantly more pronounced than in non-septic controls (Fig. 1). However, when comparing the extent of activation in COVID-19 and control patients, who had deceased under septic conditions, no difference was found (Fig. 1d). Significant activation of microglia was also found in other brain regions, including olfactory bulb and medulla oblongata in COVID-19 as well as control brains (Suppl. Figure 1). Therefore, we conclude that the microglia activation observed in COVID-19 patients represents a histopathological correlate of a critical illness-related encephalopathy, and is not a disease-specific finding.

**Fig. 1:**
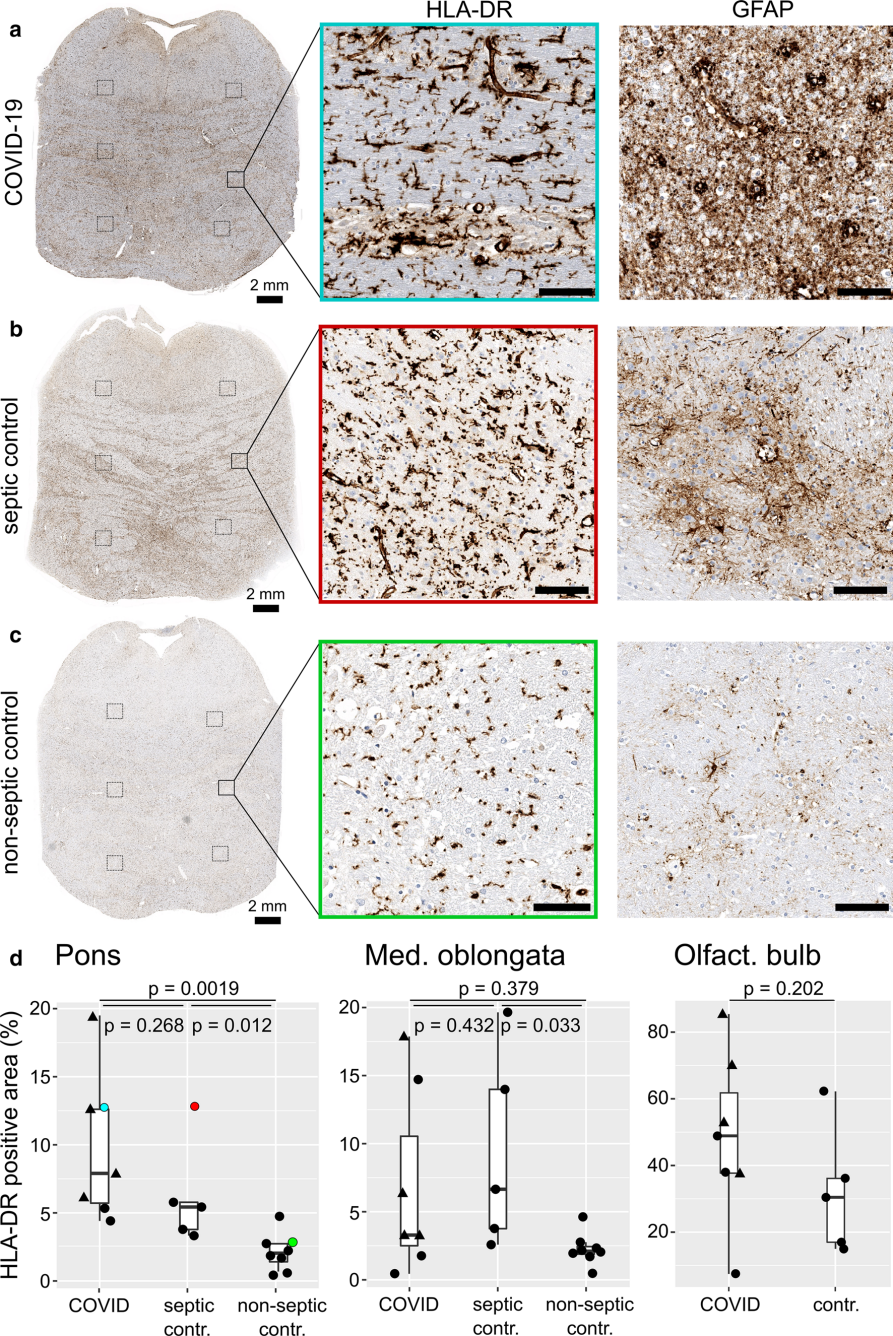
Microglia and astroglia activation in the pons. **a**–**c** Activation of microglia (stained for HLA-DR) and astrocytes (stained for GFAP). Representative sample of the COVID-19 cohort (**a**, case 4), a control with fungal sepsis and systemic inflammation (**b**, case 10), and a non-septic control (**c**, case 15). Histology image frame colors indicate individual case data points in the quantitative analysis below. **d** Automated quantification of HLA-DR immunopositive areas in the pons, medulla oblongata, and olfactory bulb. Each data point represents the mean of six crack artifact-free areas per slide and case of the COVID-19 cohort compared to controls. SARS-CoV-2 positive cases represented by triangles. Scale bars represent 100 μm unless otherwise indicated

We did not find any evidence for a COVID-19-related meningitis/encephalitis with increased lymphocytic infiltration of the brain or the leptomeninges. Although we detected sparse perivascular and leptomeningeal infiltrates of CD3^+^ T lymphocytes in some COVID-19 brains, this was similarly encountered in controls with sepsis or systemic inflammation. Likewise, in none of the brains of our study cohort, intraparenchymal hemorrhages or acute/subacute ischemic infarcts were found. Furthermore, we did not detect microthrombi or fibrinoid necrosis in intracerebral or leptomeningeal blood vessels, indicating that the pathomechanism of disseminated microthrombotic pulmonary vessel occlusions [1, 18] is not generally prominent in the brain. This is noteworthy, as a number of COVID-19 patients have been reported to develop cerebrovascular complications with ischemic stroke [3, 5, 9], perhaps reflecting altered general coagulation homeostasis [6, 10] and/or endothelial involvement [24] in severe disease. In two brains of our study cohort histopathological correlates of acute hypoxic-ischemic encephalopathy were noted—an expected finding given the prominent pulmonary impairment with consecutive hypoxia in severe COVID-19 [11, 25].

With regard to the widely accepted concept that CNS inflammation can contribute to the progression of neurodegenerative diseases [7], it is worth mentioning that one study cohort patient with Parkinson’s disease diagnosed at autopsy (case 3) did not show any exacerbation of his pre-existing extrapyramidal symptoms due to COVID-19.

Anosmia is a frequent early neurological sign of infection with SARS-CoV-2 [14]. In addition, based on observations in SARS-CoV and MERS animal models [19], neurotropic properties have been suggested for SARS-CoV-2 [4]. Recent studies suggested axonal transport of SARS-CoV-2 via the cribriform plate as a route of CNS entry [23], affecting the sense of smell by infecting olfactory bulb neurons and/or glial cells. The detection of SARS-CoV-2 RNA specifically in that particular location—but not in any other brain region—in 4/7 patients of our study cohort (Suppl. Table 4) would lend support to the postulated viral entry via the olfactory system.

As angiotensin-converting enzyme 2 (ACE2) has been identified as an entry receptor for SARS-CoV-2 [2, 17], we investigated its expression in olfactory bulb and brainstem. Whereas, by immunohistochemistry, weak ACE2 expression was found in endothelia of leptomeningeal and intracerebral blood vessels as well as in neurons of the brainstem, no expression at immunohistochemically detectable levels was found in the olfactory bulb. In contrast, spatial transcriptomics using RNAscope revealed sparse *ACE2* expression also by olfactory bulb (and brainstem) neurons, as well as by few astrocytes (Suppl. Figure 2). In particular, as the expression of ACE2 was not upregulated in COVID-19 brains when compared to controls, it remains unclear whether its upregulation plays a role in the context of COVID-19. These findings may indicate that ACE2 expression below levels detectable by immunohistochemistry is sufficient for SARS-CoV-2 entry into target cells.

Our findings represent endpoints of particularly severe disease, with single time point qRT-PCR measurements precluding conclusions about a potentially dynamic brain viral load during the course of disease. It is possible that reversible brain alterations such as reactive inflammatory processes that had potentially occurred earlier during the clinical course, went unnoticed. These limitations notwithstanding, based on our observations, it seems unlikely that irreversible changes such as acute demyelination or ischemic lesions are part of the usual spectrum of COVID-19 with brain involvement. Instead, several histological brain abnormalities previously postulated to occur in association with COVID-19 can likewise be seen in SARS-CoV-2 negative, critically ill patients. Our observations suggest that specific SARS-CoV-2 induced neuropathological abnormalities are absent in the majority of COVID-19 patients.

## Electronic supplementary material

Below is the link to the electronic supplementary material.

Supplementary file1 (PDF 36557 kb)
