# Supplementary material for: Correlates of critical illness-related encephalopathy predominate postmortem COVID-19 neuropathology
Source: Acta Neuropathol. 2020 Aug 26;140(4):583–6. doi: 10.1007/s00401-020-02213-y (PMC7449525; doi:10.1007/s00401-020-02213-y)
Supplement: Supplementary file 1 — Supplementary file1 (PDF 36557 kb) [file 401_2020_2213_MOESM1_ESM.pdf]

Supplementary information to

Correlates of critical illness-related encephalopathy predominate postmortem COVID-19 neuropathology

Nikolaus Deigendesch[1], Lara Sironi[1], Michael Kutza[2], Sven Wischnewski[2], Vidmante Fuchs[3], Jürgen Hench[1], Angela Frank[4], Ronny Nienhold[4], Kirsten D. Mertz[4], Gieri Cathomas[4], Matthias S. Matter[1], Martin Siegemund[5,6], Markus Tolnay[1], Lucas Schirmer[2], Anne-Katrin Pröbstel[3], Alexandar Tzankov[1], and Stephan Frank[1*]

[1] Pathology, Institute of Medical Genetics and Pathology, University Hospital Basel, University of Basel, Switzerland

[2] Department of Neurology and Mannheim Center for Translational Neuroscience, Medical Faculty Mannheim, University of Heidelberg, Mannheim, Germany

[3] Neurologic Clinic and Policlinic, Departments of Medicine and Biomedicine, University Hospital of Basel, University of Basel, Basel, Switzerland

[4] Institute of Pathology, Cantonal Hospital Baselland, Liestal, Switzerland

[5] Department of Intensive Care, University Hospital, University of Basel, Basel, Switzerland

[6] Department of Clinical Research, University of Basel, Switzerland

Patients and Methods

Study and control cohorts

We performed autopsies of seven brains from COVID-19 patients (study cohort; 6:1 male/female ratio) with a mean age of 71.5 years (range 54 to 96 yrs.). Of note, as we did not perform autopsies in COVID-19 patients of the oldest old group (i.e. age > 80 yrs.), our study cohort is likely only partly representative of the general population of COVID-19 deceased with regard to age [3, 11].

As control cohort, we analyzed brains of SARS-CoV-2 negative individuals (n=13) deceased during the same time period; this control cohort was further subdivided into non-septic controls (n = 8; 3:1 male/female ratio; mean age 80.5 yrs.; patients succumbed due to sudden death without systemic inflammatory response) and septic controls (n=5; 4:1 male/female ratio; mean age 72.8 yrs.; patients with severe systemic inflammatory or septic clinical course). SARS-CoV-2 status, as well as clinically relevant information for both study and control cohort patients, are summarized in Supplementary tables 1 and 2; treatment details of the study cohort are listed in Supplementary table 3. All autopsies were performed at the Institute of Medical Genetics and Pathology at the University Hospital of Basel, Switzerland. Six of the seven COVID-19 patients were part of a larger, recently reported whole body-autopsy cohort [7]. Our study was approved by the Ethics Committee of Northwestern and Central Switzerland (ID 2020-00629 and 2020-00969).

To avoid aerosolization in the case of COVID-19, brains were removed upon opening the skull with a handsaw [7]. Mean postmortem interval to brain removal was 28.2 hours (range 7 to 71 hrs.). Following *in toto* fixation in standard 4% (w/v) phosphate-buffered formalin, samples from various brain regions including several neocortical areas, cerebral white matter, hippocampus, amygdala, striatum, brainstem, medulla oblongata, cerebellum, olfactory bulb, and optic nerve were collected for each case.

Clinical observations

All but one of the COVID-19 patients suffered from hypertension or hypertensive cardiovascular disease and had multiple significant comorbidities (Suppl. table 1) known to correlate with poorer clinical outcome [4, 5, 7]. Whereas two patients (patients 5 and 7) were unconscious at

the time of hospitalization following rapid deterioration into cardiogenic shock, the other five presented with cough and flu-like symptoms at hospital admission. All patients of our study cohort were *ante mortem* diagnosed for COVID-19 using qRT-PCR from nasopharyngeal swabs (Suppl. table 4), whereas the patients of our control cohort, all of whom had multiple significant comorbidities as well, did not show clinical signs of COVID-19 and were tested negative by qRT-PCR.

Four of seven COVID-19 study cohort patients were transferred to the intensive care unit (ICU) and received assisted ventilation. Except for two patients, who did not receive antiviral treatment due to contraindications, all study cohort patients received various regimens of antiviral therapy, such as hydroxychloroquine (5/7), remdesivir (1/7), and lopinavir/ritonavir (3/7) (Suppl. table 3). One patient died immediately after admission to the emergency room. Because of suspected or confirmed secondary bacterial respiratory infection, 5/7 patients were treated with antibiotics; two of them also received systemic antifungal treatment and the monoclonal IL-6 receptor antibody tocilizumab [10]. One patient was treated with convalescent plasma derived from recovered donors [2, 6]. Except for one patient who succumbed to multiple organ failure after initial presentation with acute heart failure and cardiogenic shock, all other patients of our study cohort (6/7) died of respiratory failure (Suppl. table 2).

In one patient, who suffered from progressive multiple sclerosis (MS), no clinical aggravation of MS-related symptoms was noted in association with COVID-19, and likewise, upon histological examination, demyelinating foci of the cerebral white matter did not show any evidence of inflammation or myelophagia (not shown). In patient 3, who had a clinical history of long-standing extrapyramidal symptoms, brain autopsy revealed numerous Lewy bodies and Lewy neurites within the substantia nigra and the locus coeruleus (not shown), leading to the diagnosis of idiopathic Parkinson's disease. Also, this patient did not experience any worsening of his pre-existing neurological symptoms in association with COVID-19.

Neurological symptoms in plausible association with COVID-19 were only verified in one patient of the study cohort, who reported dizziness at disease onset. All other study cohort patients could either not be asked (intubation with analgo-sedation/neuromuscular block at time of admission) or the reported neurological symptoms (disorientation and agitation) had already

started prior to hospitalization. Unfortunately, at that time, the loss of sense of smell and taste was not systematically recorded.

Histology

Formalin-fixed paraffin-embedded (FFPE) brain tissue samples were processed following standard histochemical protocols, including hematoxylin and eosin (H&E) staining. Immunohistochemical examinations were performed for fibrinogen (Dako, A0080), CD45/LCA (RP2/18), glial fibrillary acidic protein (GFAP; EP672Y), HLA-DR (Dako, M0775), ACE2 (ab15348 Abcam), CD20 (L26), CD3 (2GV6), and alpha-synuclein (LB509) using Ventana BenchMark Ultra automated staining machine and the OptiView DAB IHC Detection Kit (Ventana, Roche) or stained manually using Stable DAB/Plus (Diagnostic Biosystems, Pleasanton, CA, USA).

Fluorescent multiplex *in situ* RNA hybridization

Multiplex *in situ* hybridization (ISH) was performed according to published protocols [9] and manufacturer’s recommendations (RNAscope multiplex fluorescent v2 assay kit, ACD Biotechne) to overcome technical difficulties such as high levels of background auto-fluorescence due to neuronal lipofuscin deposition [9]. Sequences of target probes, amplifier, and label probes are proprietary and commercially available (ACD Biotechne). Typically, target probes contain 20 ZZ probe pairs (approx. 50 bp/pair) covering 1000 bp. The following human manual RNAscope assay probes were used: *ACE2, AQP4-C2* and *SYT1-C3*. Following deparaffinization, FFPE tissue slides were stained with TSA Plus Fluorophores (Fluorescein, Cyanine3, Cyanine5) and 4′,6-diamidino-2-phenylindole (DAPI). As quality control, channel-specific negative (*DapB*) and positive ISH probes (*POLR2A*-C1, *PPIB-C2* and *UBC-C3*) were run in parallel.

Image analysis

Digital slide images were generated using a Ventana DP200 slide scanner (Roche) at maximum resolution. QuPath (v0.2.0-m12) [1] was used to annotate crack artifact-free brain regions for quantification of immunoreactivity with ImageJ [8]. Fluorescent images were taken with Leica TCS SP8 laser confocal (405/488/552/638 nm) microscope with 10x, 20x, 40x or 63x objectives; fluorescent confocal pictures are Z-stack images, unless stated otherwise. Images were

processed using Fiji ImageJ (v2.0) and exported to vector-based software (Adobe Illustrator, Affinity Designer, Inkscape) for figure generation. Data were analyzed using R (version 4.0.0) and visualized with ggplot2. Non-parametric testing was performed using a Wilcoxon rank-sum exact test with p value adjustment (Holm-Bonferroni method) for multiple hypotheses tests, with p values < 0.05 regarded as significant.

Molecular analyses

Postmortem viral load in the olfactory bulb, optic nerves, brainstem, and cerebellum was assayed by RT-qPCR. RNA from FFPE tissue was extracted using the RecoverAll Total Nucleic Acid Isolation Kit (Thermo Fisher Scientific, Waltham, MA, USA). Viral genomes were detected using TaqMan 2019nCoV Assay Kit v1 (Thermo Fisher Scientific), which targets three different viral genomic regions (ORFab1, S Protein, N Protein), and the human RNase P gene (RPPH1). Whereas virus detection in olfactory bulb and optic nerve did not show any obvious correlation with postmortem delay, we observed that, occasionally, with a long course of disease, even in severely affected tissues such as lung, no or very little virus was detectable (case 1).

Acknowledgments

The authors wish to thank Thomas Rost (Institute of Forensic Medicine, Basel University) and Ralf Schoch (Institute of Pathology, Basel University Hospitals) for their support with brain autopsies, and Caner Ercan for help with digital image analysis. We also thank Valeria Perrina, Michelle Baumann, Petra Hirschmann, Rosario Mamani, and Martin Herzig (all from the Institute of Pathology, Basel University Hospitals) for their excellent technical support. Funding was provided by the Botnar Research Centre for Child Health as part of the Fast Track Call for Acute Global Health Challenges as well as by the Swiss-European Mobility Programme (to V.F.), the Medical Faculty Mannheim, University of Heidelberg (to S.W.), the Hertie Foundation (to M.K. and L.S.), the National Multiple Sclerosis Society (FG-1902-33617, to L.S.; FG-1708-28871, to A.-K.P.), and intramural funding from the University of Basel.

Disclosure

The authors have no conflicts of interest to declare in regard to this study.

Supplementary references

1. Bankhead P, Loughrey MB, Fernández JA, Dombrowski Y, McArt DG, Dunne PD, McQuaid S, Gray RT, Murray LJ, Coleman HG, James JA, Salto-Tellez M, Hamilton PW (2017) QuPath: Open source software for digital pathology image analysis. Sci Rep 7:16878. doi: 10.1038/s41598-017-17204-5
2. Bloch EM, Shoham S, Casadevall A, Sachais BS, Shaz B, Winters JL, van Buskirk C, Grossman BJ, Joyner M, Henderson JP, Pekosz A, Lau B, Wesolowski A, Katz L, Shan H, Auwaerter PG, Thomas D, Sullivan DJ, Paneth N, Gehrie E, Tobian AA (2020) Deployment of convalescent plasma for the prevention and treatment of COVID-19. J Clin Invest 130:2757–2765. doi: 10.1172/JCI138745
3. Chen N, Zhou M, Dong X, Qu J, Gong F, Han Y, Qiu Y, Wang J, Liu Y, Wei Y, Xia J, Yu T, Zhang X, Zhang L (2020) Epidemiological and clinical characteristics of 99 cases of 2019 novel coronavirus pneumonia in Wuhan, China: a descriptive study. Lancet 395:507–513. doi: 10.1016/S0140-6736(20)30211-7
4. Guan W-J, Liang W-H, Zhao Y, Liang H-R, Chen Z-S, Li Y-M, Liu X-Q, Chen R-C, Tang C-L, Wang T, Ou C-Q, Li L, Chen P-Y, Sang L, Wang W, Li J-F, Li C-C, Ou L-M, Cheng B, Xiong S, China Medical Treatment Expert Group for COVID-19 (2020) Comorbidity and its impact on 1590 patients with COVID-19 in China: a nationwide analysis. Eur Respir J. doi: 10.1183/13993003.00547-2020
5. Kassir R (2020) Risk of COVID-19 for patients with obesity. Obes Rev 21:e13034. doi: 10.1111/obr.13034
6. Li L, Zhang W, Hu Y, Tong X, Zheng S, Yang J, Kong Y, Ren L, Wei Q, Mei H, Hu C, Tao C, Yang R, Wang J, Yu Y, Guo Y, Wu X, Xu Z, Zeng L, Xiong N, Liu Z (2020) Effect of Convalescent Plasma Therapy on Time to Clinical Improvement in Patients With Severe and Life-threatening COVID-19: A Randomized Clinical Trial. JAMA. doi: 10.1001/jama.2020.10044
7. Menter T, Haslbauer JD, Nienhold R, Savic S, Hopfer H, Deigendesch N, Frank S, Turek D, Willi N, Pargger H, Bassetti S, Leuppi JD, Cathomas G, Tolnay M, Mertz KD, Tzankov A (2020) Postmortem examination of COVID-19 patients reveals diffuse alveolar damage with severe capillary congestion and variegated findings in lungs and other organs suggesting vascular dysfunction. Histopathology. doi: 10.1111/his.14134
8. Schindelin J, Arganda-Carreras I, Frise E, Kaynig V, Longair M, Pietzsch T, Preibisch S, Rueden C, Saalfeld S, Schmid B, Tinevez J-Y, White DJ, Hartenstein V, Eliceiri K, Tomancak P, Cardona A (2012) Fiji: an open-source platform for biological-image analysis. Nat Methods 9:676–682. doi: 10.1038/nmeth.2019
9. Schirmer L, Velmeshev D, Holmqvist S, Kaufmann M, Werneburg S, Jung D, Vistnes S, Stockley JH, Young A, Steindel M, Tung B, Goyal N, Bhaduri A, Mayer S, Engler JB,

Bayraktar OA, Franklin RJM, Haeussler M, Reynolds R, Schafer DP, Rowitch DH (2019) Neuronal vulnerability and multilineage diversity in multiple sclerosis. Nature 573:75–82. doi: 10.1038/s41586-019-1404-z

10. Xu X, Han M, Li T, Sun W, Wang D, Fu B, Zhou Y, Zheng X, Yang Y, Li X, Zhang X, Pan A, Wei H (2020) Effective treatment of severe COVID-19 patients with tocilizumab. Proc Natl Acad Sci USA 117:10970–10975. doi: 10.1073/pnas.2005615117

11. Zhou F, Yu T, Du R, Fan G, Liu Y, Liu Z, Xiang J, Wang Y, Song B, Gu X, Guan L, Wei Y, Li H, Wu X, Xu J, Tu S, Zhang Y, Chen H, Cao B (2020) Clinical course and risk factors for mortality of adult inpatients with COVID-19 in Wuhan, China: a retrospective cohort study. Lancet 395:1054–1062. doi: 10.1016/S0140-6736(20)30566-3

Supplementary table 1: Patient clinical characteristics

cohort	patient case	sex	age (years)	days from COVID-19 symptom onset to death	COVID-19 related symptoms at admission	neurological symptoms related to COVID-19	relevant comorbidities / risk factors	pre-existing neurological conditions	ICU
COVID-19 study cohort	1	F	68	13	cough, fever	disorientation, agitation.	HT, obesity, A, steatosis hepatitis	chronic multiple sclerosis (treated with baclofen)	Yes
	2	M	86	12	cough, rhinitis	vertigo	therapy-associated MDS-EB1 after APL, DM type 2, HT, dyslipidemia, PC (in remission)	none	No
	3	M	96	unclear	fever, flu-like symptoms	none	coronary and valvular hypertensive heart disease, NSTEMI, hypothyroidism (substituted)	Parkinson's disease	No
	4	M	66	23	cough, fatigue, dyspnea, pre-renal failure	none	HT	none	Yes
	5	M	72	unclear	circulatory shock	none	dilatative arteriopathy, occlusive peripheral arterial disease, coronary and valvular heart disease, paroxysmal atrial fibrillation.	cerebrovascular disease (TIA)	No
	6	M	59	at least 19	cough, dyspnea	none	HT, obesity, A	none	Yes
	7	M	54	at least 15	circulatory shock with tachycardic atrial fibrillation	coma (GCS 3)	none	none	Yes
septic control cohort	8	M	90	-	-	-	bronchial asthma, aortic valve stenosis, NSTEMI type II, hypertensive and valvular heart disease, DM type 2	cerebrovascular disease, lacunar infarction in basal ganglia	Yes
	9	M	70	-	-	-	A, HT, pulmonary adenocarcinoma	cerebrovascular disease, multiple old lacunar cerebral infarctions	Yes
	10	M	64	-	-	-	MDS-EB1	none	Yes
	11	M	63	-	-	-	A, coronary heart disease, multiple myeloma with allo-HSCT, systemic AL amyloidosis, gout	none	Yes
	12	F	77	-	-	-	A, HT, plasma cell myeloma, melanoma	cerebrovascular disease, old lacunar cerebral infarction	No*
non-septic control cohort	13	M	89	-	-	-	A, coronary and valvular heart disease, chronic kidney disease	none	No
	14	M	95	-	-	-	A, obesity, coronary and valvular hypertensive heart disease, HT, ATTR amyloidosis	none	No
	15	M	79	-	-	-	A, HT, colon carcinoma (i.r.)	none	No
	16	M	77	-	-	-	coronary and valvular heart disease, severe aortic valve stenosis, myocardial infarction, A, DM type 1	cerebrovascular disease, pontine lacunar infarction	No
	17	M	83	-	-	-	therapy-associated AML after radiotherapy of PC (i.r.), HT, A, coronary heart disease, DM type 2	none	No
	18	M	65	-	-	-	A, systemic sclerosis, coronary heart disease, atypical CLL	none	No
	19	W	84	-	-	-	A, pleural mesothelioma, breast cancer (i.r.), ovarian carcinoma (i.r.)	multiple acute cerebral infarctions	No
	20	W	72	-	-	-	A, pulmonary adenocarcinoma	none	No

A, atherosclerosis
AML, acute myeloid leukemia
APL, acute promyelocytic leukemia
CLL, chronic lymphatic leukemia
COPD, chronic obstructive pulmonary disease

CRP, C-reactive protein
DM, diabetes mellitus
GCS, Glasgow coma scale
HSCT, hematopoetic stem cell transplantation
HT, arterial hypertension

ICU, intensive care unit
i.r., in remission
MDS-EB1, myelodysplastic syndrome with excess blasts
NSTEMI, non-ST-elevation myocardial infarction
PC, prostate carcinoma
TIA, transient ischemic attack

* was not admitted to the ICU due to palliative treatment

Supplementary table 2: Autopsy findings

	patient case	postmortem delay (hours)	autoptic cause of death	brain autopsy findings
COVID-19 study cohort	1	20	respiratory failure	bilateral periventricular demyelinated lesions in white matter without active resorption or inflammation
	2	26	respiratory failure	no abnormalities noted
	3	24	respiratory failure	Lewy body pathology in subst. nigra and locus coeruleus with moderate loss of dopaminergic neurons, arteriolosclerosis in basal ganglia, mild acute hypoxic-ischemic encephalopathy
	4	11	respiratory failure	no abnormalities noted
	5	67	respiratory failure	mild acute hypoxic-ischemic encephalopathy, moderate global brain edema without cerebral mass displacement, severe atherosclerosis of the basal cerebral arteries, moderate hydrocephalus ex vacuo
	6	39	respiratory failure	mild acute hypoxic-ischemic encephalopathy
	7	71	multi organ failure	no abnormalities noted
septic control cohort	8	40	cardiac failure	old lacunar infarction in basal ganglia
	9	57	aspiration pneumonia	multifocal old lacunar infarctions
	10	14	respiratory failure in fungal pneumonia	no abnormalities noted
	11	8	biliary peritonitis after cholecystectomy	acute baso-lateral infarction
	12	29	pneumonia	old lacunar infarction of the insula
non-septic control cohort	13	7	cardiac failure with bronchopneumonia	status cribrosus of basal ganglia, vascular leukencephalopathy
	14	17	acute myocardial infarction	no abnormalities noted
	15	7	acute cardiac failure	status cribrosus of basal ganglia, vascular leukencephalopathy
	16	32	cardiac failure	old lacunar infarction in pons
	17	12	myocardial infarction with aspiration pneumonia	acute hypoxic-ischemic encephalopathy, status cribrosus of basal ganglia, arteriolosclerosis
	18	27	cardiac failure	no abnormalities noted
	19	45	acute myocardial infarction	acute cerebral and cerebellar infarctions in context of paraneoplastic embolic disease
	20	51	cardiac failure	acute subarachnoid hemorrhage

Supplementary table 3: COVID-19 study cohort treatment characteristics

patient case	antiviral treatment	other systemic antiinfectives	immunomodulatory treatment	other relevant treatment
1	hydroxychloroquine	cefepime	none	none
2	lopinavir/ritonavir hydroxychloroquine	amoxicillin/clavulanic acid	none	vilanterol/fluticason inh.
3	none	none	none	none
4	lopinavir/ritonavir remdesivir hydroxychloroquine	caspofungin piperacillin+tazobactam and clarithromycin, replaced by amoxicillin/clavulanic acid, replaced by meropenem and daptomycin	tocilizumab	none
5	none	none	none	none
6	lopinavir/ritonavir hydroxychloroquine	clarithromycin amoxicillin/clavulanic acid caspofungin meropenem daptomycin	tocilizumab	dexamethasone
7	hydroxychloroquine	azithromycin, tazobactam, meropenem	none	amiodarone, reconvalescent plasma, plasma transfusions, ECMO, microaxial ventricular assist device

ECMO, extracorporeal membrane oxygenation

Supplementary table 4: RT-qPCR results for SARS-CoV-2

patient case	olfactory bulb	optic nerve	brainstem	cerebellum	lung	SARS-CoV-2 test (nasopharyngeal swab)
1	positive	positive	negative	negative	negative	positive
2	positive	positive	negative	negative	positive	positive
3	positive	n.d.	negative	negative	positive	positive
4	negative	n.d.	negative	negative	positive	positive, also positive plasma
5	negative	n.d.	negative	negative	positive	positive
6	positive	n.d.	negative	negative	positive	positive
7	negative	negative	negative	negative	n.d.	positive, also positive plasma
controls	negative*	n.d.	negative*	negative*	negative*	negative (n=13)

n.d. = not determined

* all controls negative by swab test; therefore, RT-qPCR analyses of FFPE tissue samples was restricted to 3/13 patients (all negative).

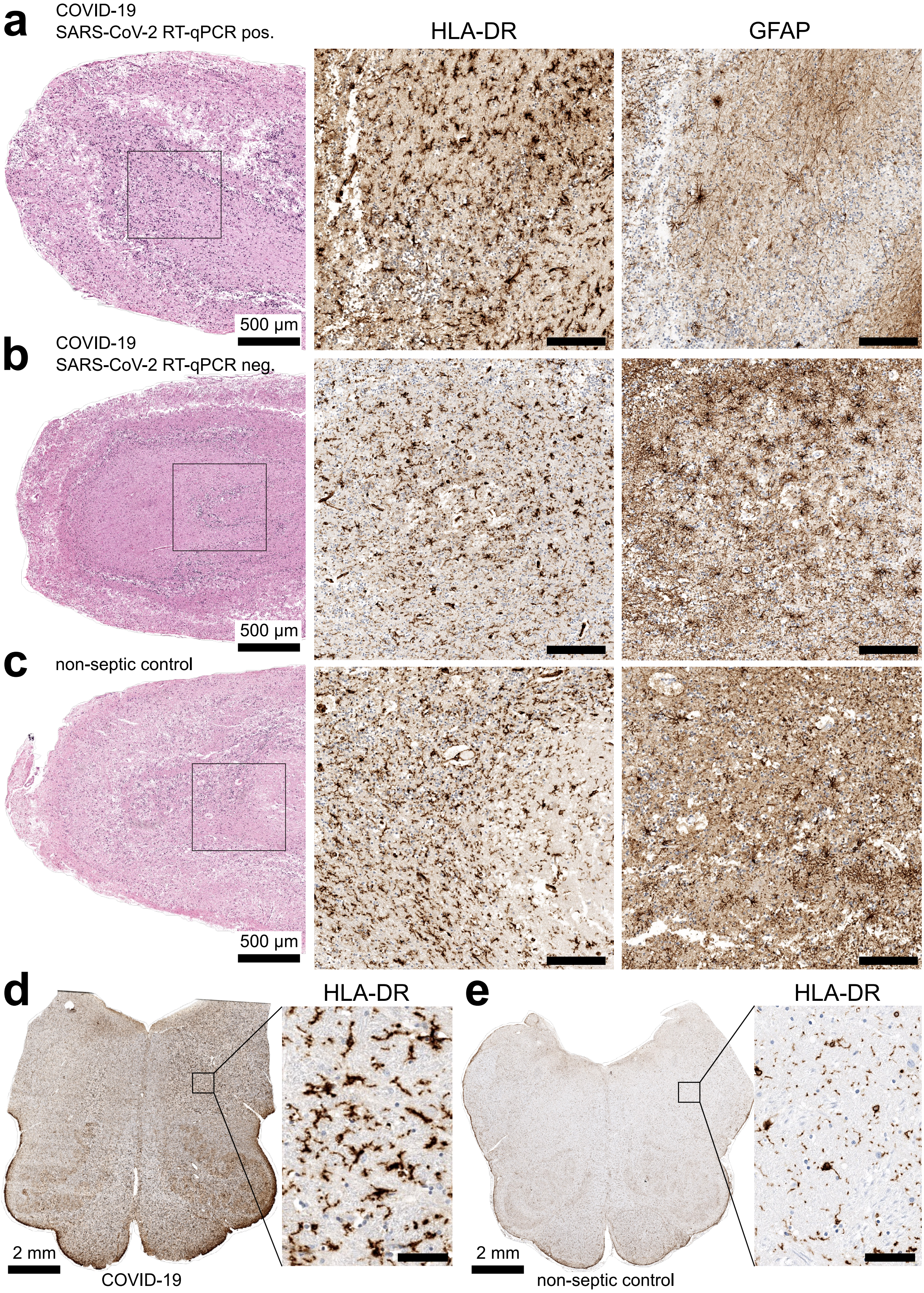

Supplementary figure 1: Microglia and astroglia activation in olfactory bulb and medulla oblongata.
(**a - c**) H&E stains and immunohistochemistry for HLA-DR and GFAP for olfactory bulb of COVID-19 patients with (**a**, case 2) or without (**b**, case 4) SARS-CoV-2 detected by post-mortem RT-qPCR, compared to non-septic control (**c**, case 15). (**d, e**) HLA-DR expression in medulla oblongata of a post-mortem SARS-CoV-2 negative COVID-19 patient (**d**, case 7) and non-septic control patient (**e**, case 13) stained for HLA-DR. Scale bars represent 100 µm unless otherwise indicated.

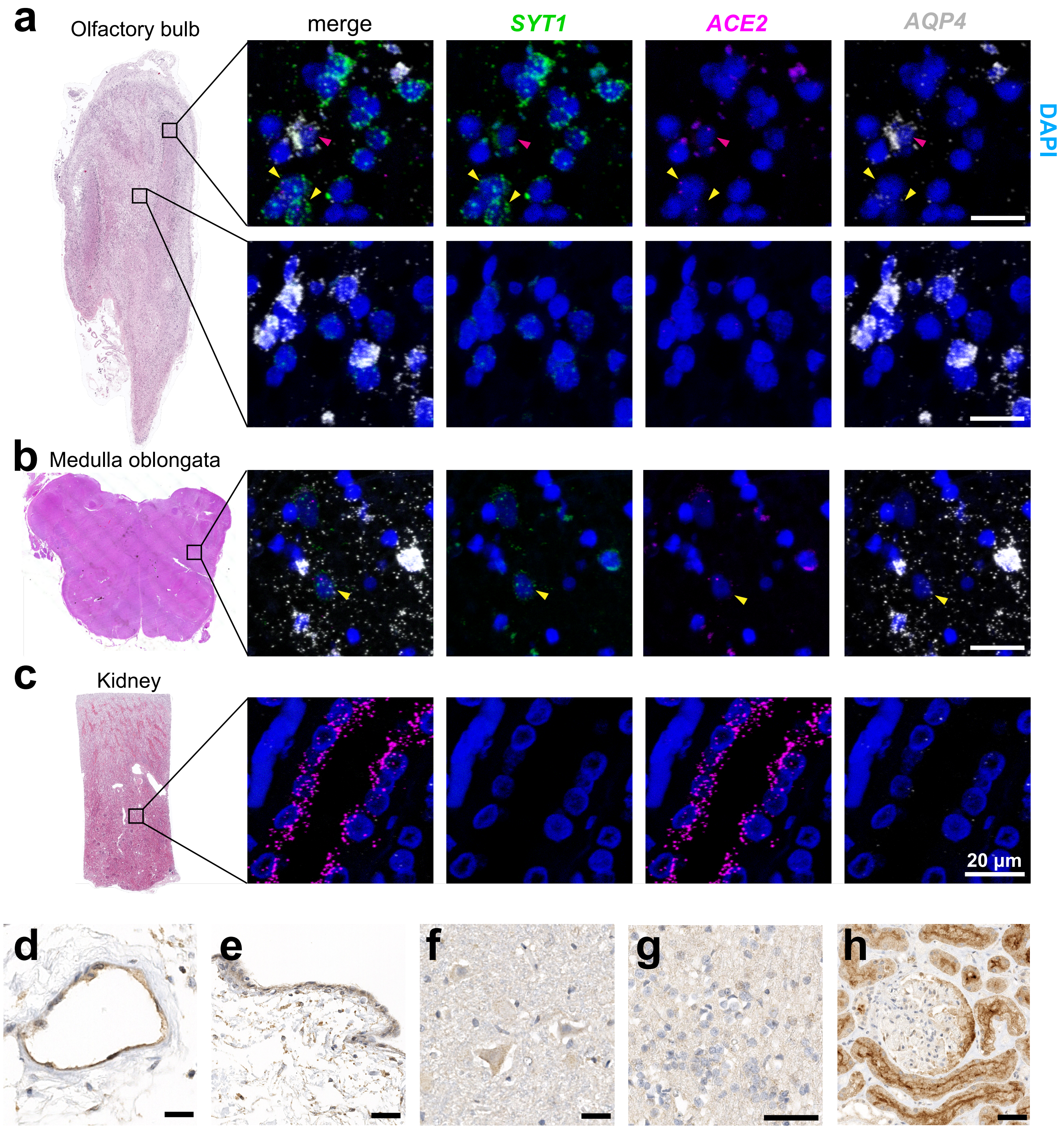

Supplementary figure 2: Tissue expression of angiotensin-converting enzyme 2 (ACE2). (**a - b**) Spatial transcriptomics showing overall sparse co-expression of synaptogamin 1 (*SYT1*, green) with *ACE2* in neuronal cells (yellow arrow heads) and aquaporin 4 (*AQP4*, grey) with *ACE2* (magenta) in glial cells (magenta arrow heads) in marked regions of the bulbus olfactorius and medulla oblongata. (**c**) Kidney tissue stained as control shows strong *ACE2* expression in tubulus epithelial cells. Nuclei are stained with DAPI (blue). Scale bars represent 20 µm. (**d - h**) Immunohistochemistry for human ACE2 (brown; counterstained with hematoxylin) reveals weak ACE2 protein expression in leptomeningeal endothelial (**d**) and meningothelial cells (**e**). (**f**) Weak ACE2 expression in neurons of the medulla oblongata. (**g**) No ACE2 expression is detectable in neurons and glial cells of the olfactory bulb by immunohistochemistry. (**h**) Kidney tubulus cells were used as positive control. Scale bars represent 50 µm.

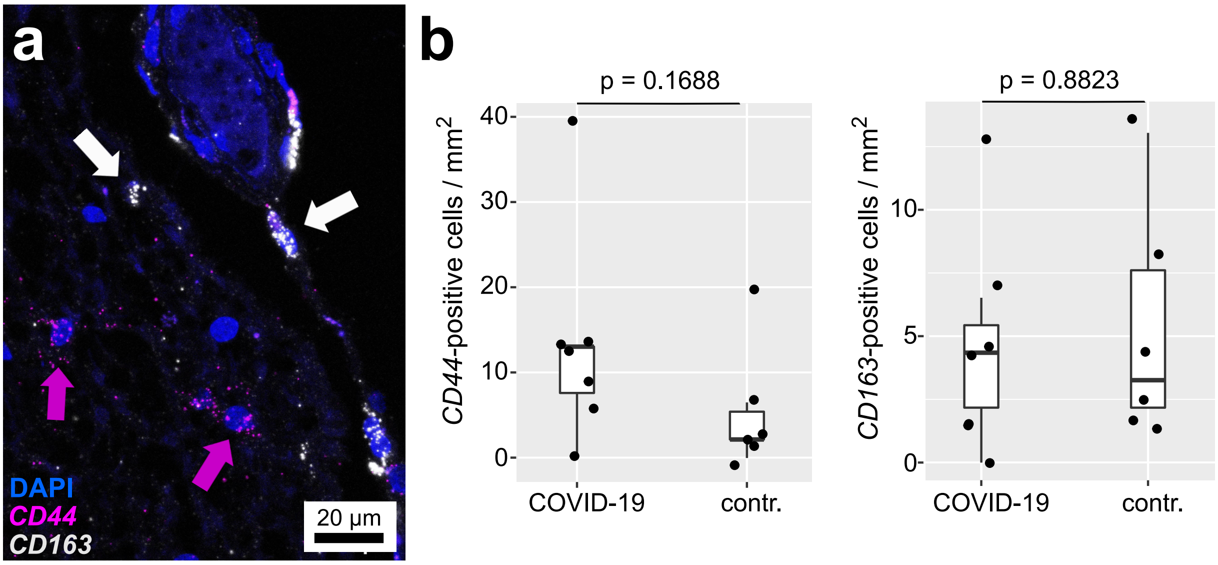

Supplementary figure 3: Spatial transcriptomics of markers of activated myeloid (*CD163*) and reactive astroglial (*CD44*) subtypes in the pons.
(**a**) *CD163*-expressing macrophages (perivascular) and microglia in the parenchyma (white arrows). *CD44*-expressing reactive astrocytes (magenta arrows) are present in perivascular space and rarely in the parenchyma of the pons (case 4). (**b**) Comparative quantification of selected ROIs in COVID-19 versus control patients reveals no difference for *CD44*-expressing astroglia (left) and *CD163*-positive myeloid cells (right). Each data point represents the mean of six crack artifact-free areas per slide and case.
